# Unlocking the Diagnostic Challenge of Tuberculosis and Sarcoidosis Intrathoracic Lymphadenopathy: Potential Role of HMGB1 and miRNA-221 as Diagnostic Tools

**DOI:** 10.3390/microorganisms14020369

**Published:** 2026-02-04

**Authors:** Fatma Z. Kamel, Nagwan Adel Ismail, Asmaa Z. Khater, Alia A. El Shahawy, Noura Almadani, Chandrakala Sankarapandian, Noha M. Hammad

**Affiliations:** 1 Department of Medical Microbiology and Immunology, Faculty of Medicine, Zagazig University, Zagazig 44519, Egypt; fzkhater@medicine.zu.edu.eg (F.Z.K.); aaelshahawy@medicine.zu.edu.eg (A.A.E.S.); 2 Chest Department, Faculty of Medicine, Zagazig University, Zagazig 44519, Egypt; naelsaid@medicine.zu.edu.eg; 3 Radiodiagnosis Department, Ahmed Maher Teaching Hospital, General Organization of Teaching Hospitals and Institutes, Cairo 11562, Egypt; asmaakamel551@gmail.com; 4 Community and Psychiatric Mental Health Nursing Department, College of Nursing, Princess Nourah bint Abdulrahman University, Riyadh 11671, Saudi Arabia; naalmadani@pnu.edu.sa; 5 Medical Surgical Nursing Department, College of Nursing, Princess Nourah bint Abdulrahman University, Riyadh 11671, Saudi Arabia; csshenbagathammal@pnu.edu.sa; 6 Al Jouf Regional Laboratory, Shared Services, Ministry of Health, Sakaka 72311, Saudi Arabia

**Keywords:** high mobility group box 1 protein, QuantiFERON-TB gold, MiRNA-221, sarcoidosis, TB

## Abstract

Tuberculosis and sarcoidosis can present with similar clinical and radiological features, especially intrathoracic lymphadenopathy, complicating differential diagnosis. This study explored the potential utility of QuantiFERON-TB Gold (QFT), serum High Mobility Group Box 1 protein (HMGB1), and microRNA-221 (miRNA-221) relative expression as biomarkers to aid in distinguishing tuberculosis-related intrathoracic lymphadenopathy (TBIL) from sarcoidosis-related intrathoracic lymphadenopathy (SAIL). The study included 27 patients with TBIL, 27 patients with SAIL, and 27 healthy controls. QFT results, serum HMGB1 levels, and miRNA-221 relative expression were measured and compared across groups using univariable and exploratory multivariable analyses. Significant differences were observed among the study groups for serum HMGB1 levels, miRNA-221 expression, and QFT results (*p* < 0.001). Both TBIL and SAIL patients had significantly higher HMGB1 levels compared with healthy controls, consistent with inflammatory activity. In contrast, miRNA-221 expression was significantly elevated in TBIL patients compared with both SAIL patients and controls. Exploratory analyses suggested a potential contribution of miRNA-221 to differentiating TBIL from SAIL, whereas the effects of HMGB1 and QFT were less pronounced after adjustment. The findings suggest that miRNA-221, alongside HMGB1 and QFT, may contribute to the differentiation of TBIL from SAIL, although validation in larger cohorts is necessary.

## 1. Introduction

Tuberculosis (TB) constitutes a major global health burden. It is a chronic infectious disease caused by the *Mycobacterium tuberculosis* (MTB) complex, with the pulmonary system representing the principal site of infection. Nevertheless, extrapulmonary involvement is frequently observed, with intrathoracic lymph nodes being the most common site of disease manifestation, predominantly affecting mediastinal and hilar lymph nodes and is classified as TB intrathoracic lymphadenopathy (TBIL). In adult populations, TBIL may present as an isolated pathology or in association with concomitant pulmonary parenchymal involvement [1].

Sarcoidosis is a chronic granulomatous disease of unknown aetiology. Patients typically present with asymptomatic lymphadenopathy with or without pulmonary infiltrations. The most common radiological finding is bilateral hilar lymphadenopathy. Mediastinal lymphadenopathy may also be found and is usually associated with pulmonary infiltrates [2]. In 2020, the American Thoracic Society introduced updated diagnostic criteria for sarcoidosis; however, certain elements remain insufficiently standardized [3]. The diagnostic framework is based on three components: the clinical manifestations, the presence of non-necrotizing granulomatous inflammation in tissue specimens, and the exclusion of alternative etiologies of granulomatous disease [4].

Pulmonary sarcoidosis and TB represent the most encountered tissue confirmed granulomatous disorders. The nature of their relationship has long been a subject of debate. Some researchers propose that TB and sarcoidosis reflect two ends of a single disease spectrum, whereas others reject any causal association between them. Nonetheless, the marked clinical similarities between the two conditions render their differential diagnosis particularly challenging [5]. In certain cases, imaging and histopathological evaluations may not reliably differentiate TB from sarcoidosis. This diagnostic uncertainty is clinically relevant, as studies have estimated that approximately 15–20% of patients with granulomatous lymphadenopathy may be misclassified when conventional diagnostic approaches are used [4].

Traditional diagnostic tools, including the tuberculin skin test (TST), QuantiFERON-TB Gold assay (QFT), serum angiotensin-converting enzyme (ACE) levels, imaging, and histopathology, show variable accuracy. For example, TST sensitivity is reduced in sarcoidosis due to anergy, while QFT exhibits 60–80% sensitivity and 90–95% specificity for latent TB infection [5,6,7]. Serum ACE is elevated in 60–80% of sarcoidosis cases, but it is not disease-specific, as TB may also increase the ACE levels [8]. Histopathology may fail to distinguish TB from sarcoidosis when caseous necrosis is absent [9]. Together, these limitations increase the risk of misdiagnosis and inappropriate therapy, particularly the use of immunosuppressants in undiagnosed TB patients.

High Mobility Group Box 1 (HMGB1) is a prototypical alarmin released from stressed or apoptotic cells during the course of TB, existing in multiple redox states. It is a non-histone nuclear protein composed of 215 amino acids, organized into two DNA binding domains (A box and B box) and a negatively charged C-terminal tail rich in glutamic and aspartic residues. The role of HMGB1 in TB immunopathogenesis is thought to influence both the regulation of inflammatory processes and the modulation of host immune responses [7].

Recent investigations have focused on the dysregulated expression of microRNAs (miRNAs) in TB and sarcoidosis, with most studies evaluating their diagnostic relevance in pulmonary disease or through the analysis of peripheral blood mononuclear cells, Broncho alveolar lavage fluid, urine, or serum compared with healthy controls. MiRNAs are small, endogenous, single stranded non-coding RNAs that regulate gene expression post-transcriptionally and play essential roles in immune regulation and granulomatous inflammation [8]. Among them, miRNA-221 has been reported to be upregulated in patients with pulmonary TB [9]. In contrast, reduced serum miRNA-221 expression has been described in sarcoidosis patients with pulmonary fibrosis, where it was suggested to exert an anti-inflammatory role [10]. However, whether miRNA-221 demonstrates a disease-specific expression pattern in patients presenting with intrathoracic lymphadenopathy and whether it can contribute to the differential diagnosis between TBIL and SAIL remain unclear. This gap underscores the need to evaluate miRNA-221 in a clinically relevant diagnostic context where TBIL and SAIL pose a significant challenge.

Given the substantial overlap in clinical and radiological features between TBIL and SAIL, together with the limited sensitivity and specificity of traditional diagnostic tools, there remains a clear unmet need for reliable, non-invasive biomarkers to improve diagnostic discrimination. Accordingly, the present study aimed to assess the serum HMGB1 levels, relative expression of miRNA-221, and QFT results in patients with TBIL and SAIL and to investigate their potential utility in distinguishing between these two conditions in patients presenting with intrathoracic lymphadenopathy.

## 2. Materials and Methods

### 2.1. Sample Size Calculation

The sample size of 27 subjects per group was calculated using OpenEpi 3.01 based on the prevalence of positive QFT in TBIL patients versus controls reported by He et al. (2023) [1] of 81.8% vs. 51.5% (targeting 80% power at 95% confidence level). Comparisons of QFT positivity rates between groups were performed using chi square (χ^2^) test resulting in a total of 81 subjects (27 subjects per group for SAIL, TBIL, and control groups) included in the study.

### 2.2. Study Design

This study adopted a case–control design and was implemented during the period from January 2024 to September 2024. Patients were recruited from Zagazig University Hospital (Zagazig, Egypt) Chest inpatient wards. In vitro investigations were performed at the Immunology and Molecular Biology Laboratories of the Medical Microbiology and Immunology Department, Faculty of Medicine, Zagazig University (Zagazig, Egypt), while radiodiagnosis of participant patients was performed at the Radiology Department of the Ahmed Maher Teaching Hospital (Cairo, Egypt).

### 2.3. Inclusion and Exclusion Criteria

The work was conducted under the Ethics code of the World Medical Association (Declaration of Helsinki) for experiments including human cases. The Institutional Review Board (IRB) Committee of the Faculty of Medicine, Zagazig University authorized the study protocol (approval no. 67/22-1-2024). Patients aged 18–60 years with TBIL or SAIL were clinically eligible and included in our study.

The inclusion criteria for this study were TBIL and SAIL patients diagnosed clinically by a specialist physician through complete medical history taking including respiratory symptoms (dry and/or productive cough, dyspnea, chest pain and hemoptysis) and thorough general and chest examination. The laboratory results were collected, and features of the high resolution computed tomography (HRCT) were carefully evaluated by a senior radiologist.

Age-and sex-matched healthy volunteers were recruited as controls. Inclusion criteria for controls included the absence of any chronic or acute illness, no history of TB or sarcoidosis, and no prior exposure to TB. Latent TB infection was excluded in all controls based on negative QFT results.

Exclusion criteria for this study included the participants’ refusal to participate, presence of active pulmonary TB, chronic inflammatory diseases such as aspergillosis and chronic hepatitis, systemic autoimmune disorders including systemic lupus erythematosus, rheumatoid arthritis, and systemic sclerosis as well as malignancies and ischemic heart disease.

### 2.4. Blood Sampling

Six mL of peripheral blood was withdrawn from each study participant and divided between the QFT and coagulating blood collection tubes. One mL of whole blood was immediately transferred to each of the three QFT collection tubes (Nil tube, MTB tube, and mitogen tube). Blood in the coagulating collection tubes was left undisturbed at room temperature for 15–30 min to allow for clotting. The clot was removed within two hours from collection by centrifuging samples at 1000–2000× *g* for 10 min. The resulting supernatant, the designated serum, was carefully aliquoted and assessed for hemolysis by measuring free hemoglobin spectrophotometrically at 414 nm (Soret band). Samples with free hemoglobin > 50 mg/dL were considered hemolyzed and excluded from further analysis [11]. Remaining serum samples were stored at −20 °C and −80 °C for the measurement of HMGB1 and miRNA-221, respectively.

### 2.5. Tuberculin Skin Testing

TST was performed on the flexor surface of forearm according to Mantoux method [12]. Five tuberculin units (TU) of purified protein derivative (PPD) (PPD RT23; Arkray, Mumbai, India) were injected intradermally, and the injected site was marked. The injected site was evaluated for induration within 48–72 h. The results were interpreted as previously discussed [12].

### 2.6. QuantiFERON-TB Gold Test

QFT was performed according to the manufacturer’s instructions (Qiagen, Hilden, Germany). QFT collection tubes (Nil tube, MTB tube, mitogen tube) were incubated for 16–24 h at 37 °C and then centrifuged. Plasma supernatants were collected and frozen at –20 °C until measurements. After thawing, the samples were transferred into 96-wells plate and tested for the presence of interferon gamma (IFN-γ) by ELISA. The results were interpreted as positive, negative, or indeterminate [13].

### 2.7. Serum HMGB1 Level Measurement

Serum HMGB1 level was measured using sandwich ELISA commercially available kits (Cloud-Clone HMGB1, Wuhan, China) according to the manufacturer’s instructions. The microplate provided with the kit assay was precoated with the antibody specific to HMGB1. The concentration of HMGB1 in the samples was determined by comparing the optical density of the samples to the standard curve. The measurement range of the used assay was 62.5–4000 pg/mL [14].

### 2.8. MiRNA-221 Serum Level Measurement

Reverse transcription polymerase chain reaction (qRT-PCR) was used to measure the relative expression of miRNAs 221 in the serum. Total RNA was extracted from the serum using miRNeasy kits according to the manufacturer’s instructions (Qiagen, Germany, catalog no. 217004). cDNA was prepared directly from 2 μL of total RNA eluate using a 10 µL reaction of the miRCURY LNA RT Kit (Qiagen, Germany) following the manufacturer’s instructions. Although the kit is designed for standard 20 µL reactions, it can be used with volumes ranging from 10–80 µL; therefore, all reagents were proportionally scaled according to the manufacturer’s instructions. The reverse transcription reaction mixture was prepared in a final volume of 10 µL by adding the following ingredients: 2 μL 5× miRCURY SYBR^®^ Green RT Reaction Buffer, 1 μL 10× miRCURY RT Enzyme Mix, 2 μL Template RNA, and 5 μL RNase-free water. The reaction mixture was initially incubated for 60 min at 42 °C in a Rocker Sahara 320 dry bath heat block followed by a second incubation for 5 min at 95 °C to inactivate miRCURY RT Enzyme. The reaction mixture was then immediately placed on ice and stored at −80 °C.

MiRNA-221 was detected using a miRCURY SYBR^®^Green PCR Kit (Qiagen, Germany) and quantified on the “Quant studio 5 DX” platform (Thermo Fisher Scientific, Singapore).

Two separate tubes were prepared for each sample, one for miRNA-221 and the other for the U6 snRNA as a normalization gene, for the later assessment of miRNA-221 expression.

Real-time PCR reaction mixture was prepared in a final volume of 10 μL by adding the following ingredients: 5 μL 2× miRCURY SYBR Green master mix, 0.05 μL ROX reference dye, 3 μL cDNA, 1 μL PCR primer mix, and 1 μL RNase free water. The following Qiagen miRCURY LNA primer sets miRNA-221 were used: hsa-miR-221-3p (miRCURY LNA miRNA PCR Assay, Cat. No. YP00204532), and U6 snRNA (miRCURY LNA miRNA PCR Assay, Cat. No. YP00203907).

After an initial activation step for 2 min at 95 °C to activate HotStar Taq DNA polymerase, the real-time PCR reaction was run for 40 cycles of denaturation and combined annealing/extension for 10 s at 95 °C and 60 s at 56 °C, respectively.

MiRNA-221 expression was measured for the TBIL, SAIL and control samples using the previous steps to measure the cycle threshold values (*Ct*). Relative expression of miRNA-221 was calculated using the 2^−ΔΔ*Ct*^ method, where Δ*Ct* = *Ct* (miRNA-221) − *Ct* (U6) and ΔΔ*Ct* = Δ*Ct* (sample) − mean Δ*Ct* (control) [15]. All miRNA measurements were performed with the investigators blinded to the clinical diagnosis of the samples.

### 2.9. Other Laboratory Investigations and High-Resolution Computed Chromatography

Serum total and 24-urine calcium (Cobas^®^ c8000 701, Roche Cobas, Indianapolis, IN, USA) as well as angiotensin converting enzyme (ACE) (ACE kinetic, Bühlmann diagnostics, Schönenbuch, Switzerland) were measured. Bronchoscopy and transbronchial needle biopsy from enlarged lymph nodes with histopathological analysis was conducted.

High-resolution computed tomography (HRCT) was performed using a 16-slice scanner (GE 128 multislice CT, GE HealthCare, Waukesha, WI, USA) with 1 mm slice thickness and a high spatial frequency algorithm for image reconstruction. Images were assessed in both lung and mediastinal windows. Lymph nodes were categorized as either hilar or mediastinal, with enlargement defined by a maximum short axis diameter (MSAD) exceeding 10 mm. Pulmonary parenchymal opacities were classified as nodules—further subdivided into micronodules (1–4 mm) and macronodules (>5 mm)—reticular patterns, fibrotic lesions, ground glass opacities, and consolidations. Nodule distribution was further characterized as perilymphatic, centrilobular, or random [16].

### 2.10. Statistical Analysis

The normality of quantitative variables was assessed using the Shapiro–Wilk test. Comparisons between two groups were performed using the Student’s *t*-test, whereas comparisons among more than two groups were conducted using one-way ANOVA. When ANOVA indicated significant differences, the Least Significant Difference (LSD) test was applied for post hoc pairwise comparisons. Pearson’s correlation coefficient was used to evaluate the relationship between serum HMGB1 levels and relative expression of miRNA-221 in TBIL and SAIL patients. Categorical variables were analyzed using the χ^2^ or Fisher exact tests, as appropriate. Receiver operating characteristic (ROC) curve analysis was performed as an exploratory evaluation of the diagnostic performance of HMGB1 and miRNA-221, and 95% confidence interval (CI) for area under the curve (AUC), sensitivity, and specificity were calculated. A *p*-value ≤ 0.05 was considered statistically significant. All analyses were performed using SPSS version 23 (Chicago, IL, USA).

Multivariable analysis was initially attempted using standard binary logistic regression; however, the model did not converge because of the small sample size and quasi-complete separation. Therefore, Firth’s penalized likelihood logistic regression was applied. The multivariable model included ACE, HMGB1, miRNA-221, and QFT.

## 3. Results

### 3.1. Baseline Characteristics of the Study Population

The present study included a total of 81 participants, divided equally into three groups: 27 patients diagnosed with TBIL group, 27 patients diagnosed with SAIL group, and 27 healthy controls. The mean age was comparable across groups (*p* = 0.331), and the gender distribution showed no significant variation (*p* = 0.811). The baseline assessment of the study participants is presented in Table 1.

### 3.2. Clinical, Laboratory, and Radiological Characteristics of TBIL and SAIL Patients

**Figure 1:**
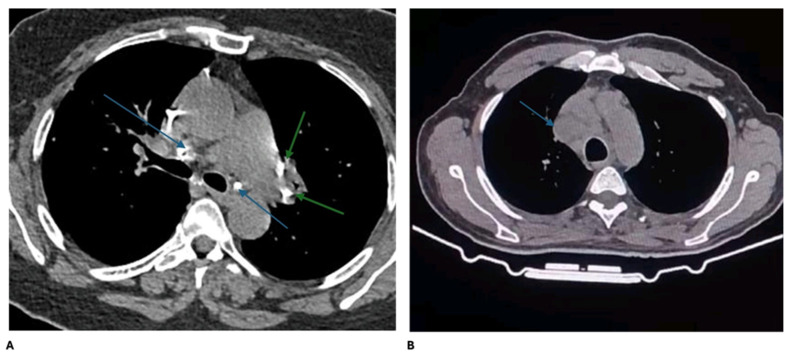
(**A**) CT chest mediastinal window revealed multiple calcified hilar and mediastinal LNS (green arrow refers to the left hilar lymph node and blue arrow refer carinal, subcarinal and aortopulmonary lymph nodes) suspected as TBIL. (**B**) CT chest mediastinal window revealed multiple pathological enlarged RT paratracheal lymph nodes with no matrix calcification (blue arrow refers to enlarged lymph nodes) diagnosed as sarcoidosis by histopathological examinations.

As demonstrated in Table 2, laboratory, radiologic, and histopathological characteristics were compared between the TBIL and SAIL groups to explore distinguishing features. Significant differences were observed between the SAIL and TBIL groups in the CBC findings and QFT results. Leucopenia/lymphopenia was more common in SAIL patients, while leukocytosis/lymphocytosis was observed only in TBIL patients (*p* = 0.002). QFT positivity was significantly higher in the TBIL patients (*p* < 0.001). Serum ACE levels were markedly elevated in the SAIL patients compared to the TBIL patients (*p* < 0.001). No statistically significant differences were found in the serum or urinary calcium levels, chest CT findings (Figure 1), biopsy categories, or TST results.

### 3.3. Serum HMGB1 Levels and miRNA-221 Relative Expression

**Figure 2:**
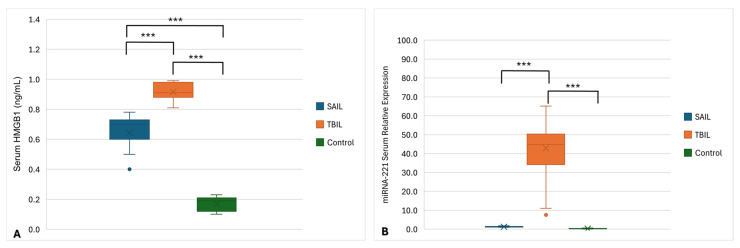
Boxplots showing the analysis of (**A**) serum HMGB1 level and (**B**) miRNA-221 relative expression among TBIL patients, SAIL patients, and control groups. Data were analyzed by ANOVA test and significant difference was defined as *** *p* < 0.001. ×, mean.

Levels of serum HMGB1 and miRNA-221 relative expression were quantitatively assessed in all participants, as shown in Figure 2. Analysis of variance (ANOVA) revealed highly significant differences in both the serum HMGB1 level and miRNA-221 relative expression among the three groups (*p* < 0.001 for both). Post hoc analysis showed that the serum HMGB1 levels were significantly higher in both disease groups compared to the controls, with TBIL patients showing the highest levels. All pairwise comparisons for HMGB1 were statistically significant (*p* < 0.001). In contrast, serum miRNA-221 was significantly elevated in TBIL patients compared to both the SAIL patients and controls (*p* < 0.001), while no significant difference was found between the SAIL and control groups (*p* = 0.667).

### 3.4. Correlation Between Serum HMGB1 and miRNA-221

**Figure 3:**
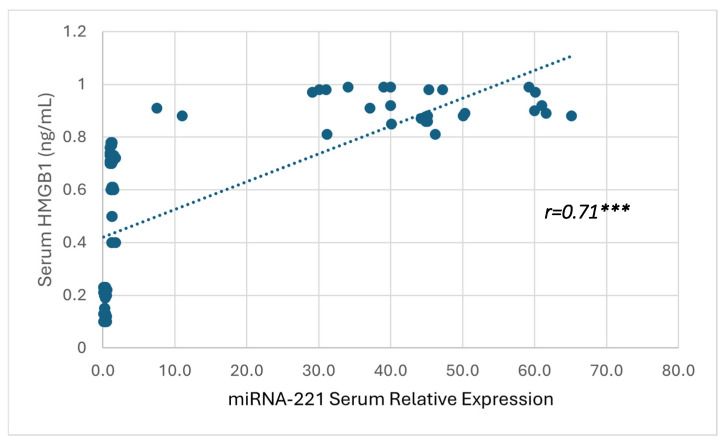
Correlation between the serum HMGB1 level and miRNA-221 relative expressions. Correlation was measured by Pearson correlation coefficient (r) and significant difference was defined as *** *p* < 0.001.

Furthermore, a strong positive correlation was observed between the serum miRNA-221 expression and HMGB1 levels (*r* = 0.71, *p* < 0.001), indicating that increased expression of miRNA-221 is significantly associated with elevated HMGB1 concentrations, as demonstrated in Figure 3.

### 3.5. Diagnostic Performance of HMGB1 and miRNA-221

**Figure 4:**
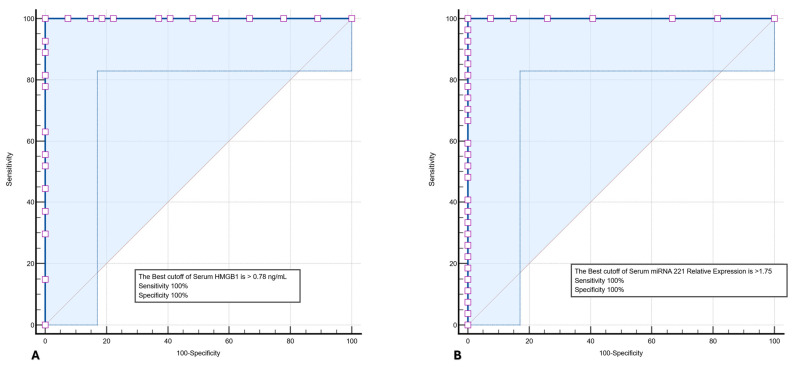
ROC curve analysis demonstrating the diagnostic performance of (**A**) serum HMGB1 level and (**B**) miRNA-221 relative expression for distinguishing TBIL from SAIL patients. AUC, sensitivity, and specificity are shown with the corresponding 95% confidence interval.

To assess the diagnostic utility of serum HMGB1 level and miRNA-221 relative expression, ROC curve analysis was performed for both biomarkers. As shown in Figure 4A, HMGB1 demonstrated an AUC of 1.00 (95% CI: 0.92–1.00), with a sensitivity of 95% (95% CI: 87.23–100%) and specificity of 100% (95% CI: 87.23–100%) at an optimal cutoff value > 0.78 ng/mL. Similarly, miRNA-221 relative expression showed an AUC of 1.00 (95% CI: 0.92–1.00), with a sensitivity of 100% (95% CI: 87.23–100%) and specificity of 100% (95% CI: 87.23–100%) at an optimal cutoff > 1.75 (Figure 4B). Given the small sample size, these results should be interpreted as exploratory, and validation in larger, independent cohorts is required to confirm the diagnostic performance of these biomarkers.

### 3.6. Multivariable Firth Penalized Logistic Regression Analysis

In multivariable Firth’s penalized logistic regression, miRNA-221 retained a modest association with TBIL versus SAIL (adjusted OR 1.11, 95% CI 1.00–2.80). ACE and QFT did not show significant adjusted associations. HMGB1 demonstrated marked group separation; however, the adjusted effect estimate was highly unstable with extremely wide confidence intervals, reflecting quasi-complete separation. These findings should therefore be interpreted as exploratory (Supplementary Table S1).

## 4. Discussion

Differentiating TBIL from SAIL remains a major diagnostic challenge because of the substantial overlap in clinical presentation, radiological features, and histopathological findings [17]. Accurate distinction is essential, as misclassification may lead to inappropriate immunosuppressive therapy in patients with unrecognized TB, with serious individual and public health consequences. In the present study, we demonstrate that while conventional clinical, radiological, and histopathological parameters showed limited discriminatory value, a combination of immunologic, hematologic, and circulating molecular biomarkers provided complementary information that improved differentiation between TBIL and SAIL.

In our cohort, histopathological evaluation did not significantly differentiate TBIL from SAIL, consistent with the shared granulomatous architecture observed in both diseases. Although granuloma morphology differs mechanistically—caseous necrosis being more typical of TB and usually absent in sarcoidosis—non-necrotizing granulomas alone cannot reliably distinguish between these entities [18,19]. This limitation is reflected in our data, where biopsy categories showed no significant association with the final clinical diagnosis, underscoring the need for adjunctive, non-invasive diagnostic tools.

Hematological parameters emerged as one such adjunct. We observed a significantly higher prevalence of leukopenia and lymphopenia among SAIL patients, whereas leukocytosis and lymphocytosis were confined to the TBIL group. These findings are in agreement with previous observations of elevated leukocyte counts in TBIL compared with SAIL [1], while cytopenias are frequently reported in sarcoidosis [20]. At the same time, the heterogeneous hematological abnormalities observed in TB patients, including both cytoses and cytopenias, align with prior reports describing the variable immune response in TB [21]. Collectively, these results support the role of basic hematologic profiles as accessible, supportive indicators rather than standalone diagnostic markers.

Serum ACE levels demonstrated a clear discriminatory trend in our study, with SAIL patients exhibiting approximately twofold higher levels compared with TBIL patients. This finding is consistent with the known expression of ACE by epithelioid and multinucleated giant cells within sarcoid granulomas and its association with granuloma burden [22]. While elevated ACE levels have been proposed as markers of disease activity and therapeutic response [23], their interpretation remains complex due to influences from lesion distribution, ACE gene I/D polymorphisms, and ethnic variability [22]. Importantly, although ACE elevation is not exclusive to sarcoidosis and may occur in TB [24], sarcoidosis remains the most common cause of markedly increased ACE levels [23], supporting its continued use as a supportive—though nonspecific—biochemical marker.

Immunologic testing revealed divergent performance between TST and IGRA in our cohort. While TST results did not differ significantly between the TBIL and SAIL patients, QFT positivity was markedly higher in the TBIL group. The lack of discriminatory value of TST in our study is consistent with the well-described “immunological paradox” of sarcoidosis, characterized by systemic anergy despite localized immune activation [25]. This phenomenon, potentially driven by impaired dendritic cell function or enhanced regulatory T-cell activity, frequently results in negative delayed-type hypersensitivity responses [26]. Accordingly, a positive TST in suspected sarcoidosis warrants careful exclusion of TB [27], but a negative result does not reliably differentiate the two conditions.

In contrast, the significantly higher QFT positivity observed in TBIL patients supports the utility of IGRAs as supplementary diagnostic tools in this context. The development of QFT, based on MTB-specific antigens such as ESAT-6 and CFP-10, has improved the specificity compared with TST [28]. Previous studies have reported the superior performance of QFT over TST for identifying latent TB infection in sarcoidosis patients [29], particularly when immunosuppressive therapy is anticipated. Nevertheless, as highlighted by WHO analyses, both TST and IGRA exhibit limited positive predictive value in high burden settings, although their negative predictive value remains consistently high [30]. Our findings reinforce the role of QFT as an adjunct rather than a definitive diagnostic test, particularly in extrapulmonary TB.

While QFT provides useful adjunctive information, real-world differentiation between TBIL and SAIL often relies on invasive procedures such as endobronchial ultrasound (EBUS)-guided lymph node biopsy or molecular testing with Xpert MTB/RIF. Xpert demonstrates high specificity (97.8–100%) and minimal laboratory risk, with lower cross-contamination rates compared with smear microscopy or culture [31]. However, its sensitivity in extrapulmonary TB is limited (32.7–66.7%) due to challenges in obtaining adequate lymph node specimens and the paucibacillary nature of the disease [32,33]. As such, a negative Xpert result does not reliably exclude TB, whereas a positive result strongly supports the diagnosis, serving effectively as a “rule-in” test [34]. In this context, circulating biomarkers such as HMGB1 and miRNA-221 could provide a non-invasive supplement to stratify patients for further diagnostic evaluation.

Beyond traditional immunologic and biochemical markers, the circulating HMGB1 and miRNA-221 levels in our study clearly distinguished TBIL from SAIL. Serum HMGB1 levels were significantly elevated in both disease groups compared with the controls, with the highest concentrations observed in TBIL patients. This gradient aligns with previous reports indicating increased HMGB1 levels in QFT-positive individuals and pulmonary TB [35]. Extracellular HMGB1 acts as a potent alarmin, amplifying inflammatory responses through interactions with receptors such as RAGE, TLR2, TLR4, and TREM-1 [35]. In TB, HMGB1 elevation likely reflects both passive release from necrotic cells—favored by MTB-induced necrosis—and active secretion by activated monocytes and macrophages [36,37]. Conversely, differential receptor expression, including higher TREM-1 levels in sarcoidosis, may enhance HMGB1 binding and up-take, potentially contributing to lower circulating levels compared with TBIL [35]. Importantly, in the context of clinical practice, HMGB1 offers a relatively long half-life and a wider diagnostic window compared with many other pro-inflammatory cytokines [35]. While our findings remain exploratory, the observed HMGB1 gradient between TBIL and SAIL provides a rationale for further validation in larger, independent cohorts to assess its utility as a non-invasive biomarker in granulomatous diseases.

In our study, miRNA-221 was significantly upregulated in TBIL patients, whereas the SAIL patients exhibited only a non-significant increase compared with the healthy controls. This pattern partially aligns with previous reports showing increased circulating miRNA-221 in pulmonary TB [38] but contrasts with studies reporting reduced miRNA-221 levels in sarcoidosis, particularly in fibrotic disease phenotypes [10]. The modest elevation observed in SAIL may reflect systemic immune activation or subclinical inflammatory processes not captured in prior studies that primarily analyzed pulmonary tissue or bronchoalveolar lavage fluid [39]. Variations in disease stage, tissue distribution, or population characteristics could also contribute to these discrepancies. Additionally, miRNA-221 and miRNA-222 are known to regulate immune responses and granuloma formation as well as promote cellular survival and proliferation via suppression of tumor suppressor genes such as PTEN and TIMP3 [40,41]. These findings underscore the potential of serum miRNA-221 as a non-invasive biomarker for TBIL while highlighting the need to interpret its levels cautiously in sarcoidosis, providing a rationale for combining it with complementary markers such as HMGB1 in diagnostic strategies.

Notably, we observed a strong positive correlation between serum HMGB1 levels and miRNA-221 expression, suggesting coordinated activation of inflammatory pathways rather than a direct mechanistic interaction. This association supports the concept that miRNA-221 may reflect broader inflammatory and necrotic processes characteristic of TBIL, contributing to elevated HMGB1 levels. However, direct regulatory interactions between miRNA-221 and HMGB1 remain speculative and warrant further investigation [42,43].

ROC curve analysis demonstrated near-perfect discrimination of TBIL from SAIL using both HMGB1 and miRNA-221. While these findings underscore the potential diagnostic value of circulating molecular markers, they must be interpreted with caution. The small sample size and marked group separation raise the possibility of overfitting, as reflected by quasi-complete separation in multivariable Firth penalized regression, particularly for HMGB1. Notably, a small subset of patients exhibited atypical or “gray-zone” results—including TB patients who were TST/QFT-negative and sarcoidosis patients who were TST-positive. In these cases, HMGB1 and miRNA-221 levels largely aligned with the clinical diagnosis relative to ROC-derived cutoffs, suggesting that these biomarkers may provide complementary diagnostic information when conventional tests are inconclusive, and are therefore best regarded as hypothesis-generating rather than definitive evidence of clinical utility.

Several limitations should be acknowledged. Despite a priori sample size calculation, the cohort remains limited for robust estimation of diagnostic accuracy and multivariable modeling. In addition, the absence of sarcoidosis patients with true concurrent TB precluded the evaluation of biomarker performance in this rare but clinically important scenario. Furthermore, the lack of external validation limits the generalizability of the observed diagnostic performance, underscoring the need for confirmation in larger, independent cohorts. Nonetheless, our findings provide integrative evidence that combining immunologic assays, hematologic profiles, and circulating molecular biomarkers—rather than relying on any single modality—may enhance diagnostic confidence in patients with intrathoracic lymphadenopathy.

## 5. Conclusions

In this exploratory study, we observed that serum HMGB1 and miRNA-221 levels exhibited distinct patterns in TBIL and SAIL patients, with HMGB1 significantly elevated in TBIL and miRNA-221 showing a TB-specific increase. These findings suggest that combining these circulating biomarkers may provide a promising, non-invasive approach to assist in the differential diagnosis of TBIL and SAIL, particularly when conventional diagnostic tests are inconclusive. However, the high sensitivity and specificity observed in this cohort should be interpreted with caution due to the limited sample size, and further validation in larger, independent multi-center cohorts is warranted. While our results indicate a potential association between miRNA-221 expression and HMGB1 levels, the study design does not allow us to infer a mechanistic or causal relationship, and this interaction should be explored in future mechanistic studies. Overall, this study provides a foundation for subsequent research aimed at validating serum biomarkers, exploring their biological underpinnings, and ultimately developing more accurate, non-invasive diagnostic tools to improve patient management in granulomatous diseases.

## Figures and Tables

**Table 1:** Baseline characteristics of the study participants.

Variables	SAIL Patients *n* = 27	TBIL Patients *n* = 27	Controls *n* = 27	Test	*p*-Value
Age (Y)				F	0.331
Mean ± SD	47.7 ± 10.5	45.9 ± 13.3	43.0 ± 11.5		
Range	25–69	19–72	24–64		
Gender				χ^2^	0.811
Female	9 (33.3)	11 (40.7)	11 (40.7)		
Male	18 (66.7)	16 (59.3)	16 (59.3)		

F; ANOVA test, χ^2^, Chi-square test.

**Table 2:** Clinical, laboratory and radiologic characteristics of SAIL and TBIL patients.

Variables	SAIL Patient *n* = 27	TBIL Patient *n* = 27	Test	*p*-Value
CBC			χ^2^	0.002 *
Leukopenia/Lymphopenia	17 (63.0)	7 (25.9)		
Leukocytosis/Lymphocytosis	0 (0.0)	8 (29.6)		
Normal lymphocyte count	10 (37.0)	12 (44.4)		
Tuberculin test			χ^2^	0.935
Positive	6 (22.2)	17 (63.0)		
Negative	21 (77.8)	10 (37.0)		
QuantiFERON-TB gold			Fisher	<0.001 *
Positive	2 (7.4)	15 (55.6)		
Negative	25 (92.6)	12 (44.4)		
Chest CT			χ^2^	0.083
Calcified hilar and mediastinal lymphadenopathy	6 (22.2)	12 (44.4)		
Non-calcified hilar and mediastinal lymph adenopathy	21 (77.8)	15 (55.6)		
Biopsy			Fisher	0.348
Caseating granuloma	2 (7.4)	4 (14.8)		
Epithelioid non-caseating granuloma	5 (18.5)	2 (7.4)		
Fibrotic specimen	4 (14.8)	8 (29.6)		
Inflammatory specimen	16 (59.3)	13 (48.2)		
Serum calcium			*t*-test	0.154
Mean ± SD	9.9 ± 0.95	9.4 ± 1.14		
Median (range)	10.2 (8.0–12.0)	9.5 (7.0–12.0)		
Urinary calcium			*t*-test	0.857
Mean ± SD	6.3 ± 1.68	6.2 ± 2.54		
Median (range)	6.1 (3.5–9.0)	6.0 (2.5–10.0)		
ACE			*t*-test	<0.001 *
Mean ± SD	148.1 ± 60.30	70.5 ± 27.86		
Median (range)	125.5 (95.0–350.0)	70.0 (23.5–110.0)		

χ^2^; Chi-square test, *t*; Student’s *t*-test. * Significant difference.

## Data Availability

The original contributions presented in the study are included in the article/Supplementary Materials, further inquiries can be directed to the corresponding author.

## Supplementary Materials

The following supporting information can be downloaded at: https://www.mdpi.com/article/10.3390/microorganisms14020369/s1, Table S1: Multivariable Firth penalized logistic regression for TBIL vs SAIL. Figure S1: Distributions of serum HMGB1 levels in patients with sarcoidosis-associated intrathoracic lymphadenopathy (SAIL), tuberculosis intrathoracic lymphadenopathy (TBIL), and healthy controls. Each dot represents one individual. Groups are color-coded for clarity (TBIL = blue, SAIL = orange, Control = gray). Figure S2: Distributions of miRNA 221 Serum Relative Expression in patients with sarcoidosis-associated intrathoracic lymphadenopathy (SAIL), tuberculosis intrathoracic lymphadenopathy (TBIL), and healthy controls. Each dot represents one individual. Groups are color-coded for clarity (TBIL = blue, SAIL = orange, Control = gray).
