# Supplementary material for: Unlocking the Diagnostic Challenge of Tuberculosis and Sarcoidosis Intrathoracic Lymphadenopathy: Potential Role of HMGB1 and miRNA-221 as Diagnostic Tools
Source: Microorganisms. 2026 Feb 4;14(2):369. doi: 10.3390/microorganisms14020369 (PMC12942768; doi:10.3390/microorganisms14020369)
Supplement: Supplementary file 1 [file microorganisms-14-00369-s001.zip › microorganisms-4091968-supplementary.pdf]

Supplementary Table S1. Multivariable Firth penalized logistic regression for TBIL vs SAIL

Variable	Adjusted OR	95% CI♠	p-value
ACE	0.99	0.80–1.08	0.651
HMGB1	48.22	$0.00–8.65\times10^{13}$	0.605
miRNA-221	1.11	1.00–2.80	0.028*
QFT (Positive vs Negative)	3.28	0.01–312,450.57	0.398

♠Firth's penalized logistic regression was used due to small sample size and separation. Some estimates show extremely wide confidence intervals and should be interpreted with caution.

*Significant difference

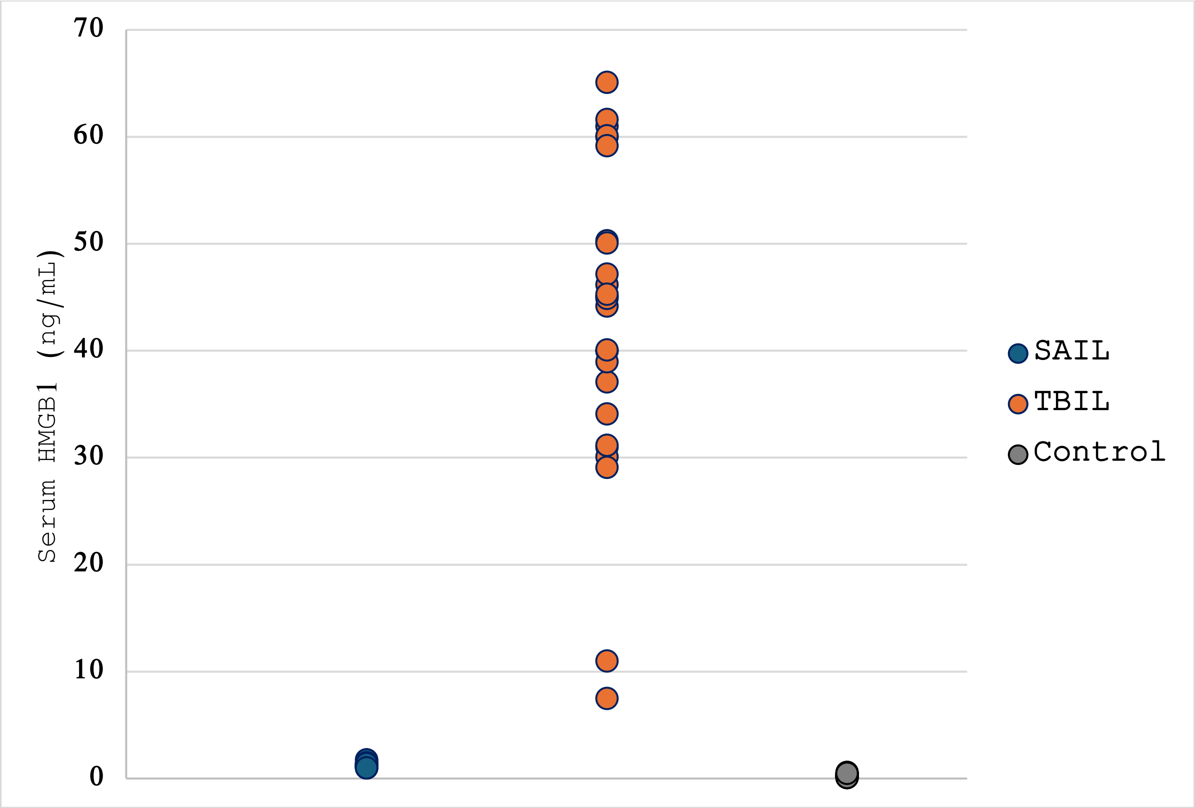

Supplementary Figure S1: Distributions of serum HMGB1 levels in patients with sarcoidosis-associated intrathoracic lymphadenopathy (SAIL), tuberculosis intrathoracic lymphadenopathy (TBIL), and healthy controls. Each dot represents one individual. Groups are color-coded for clarity (TBIL = blue, SAIL = orange, Control = gray).

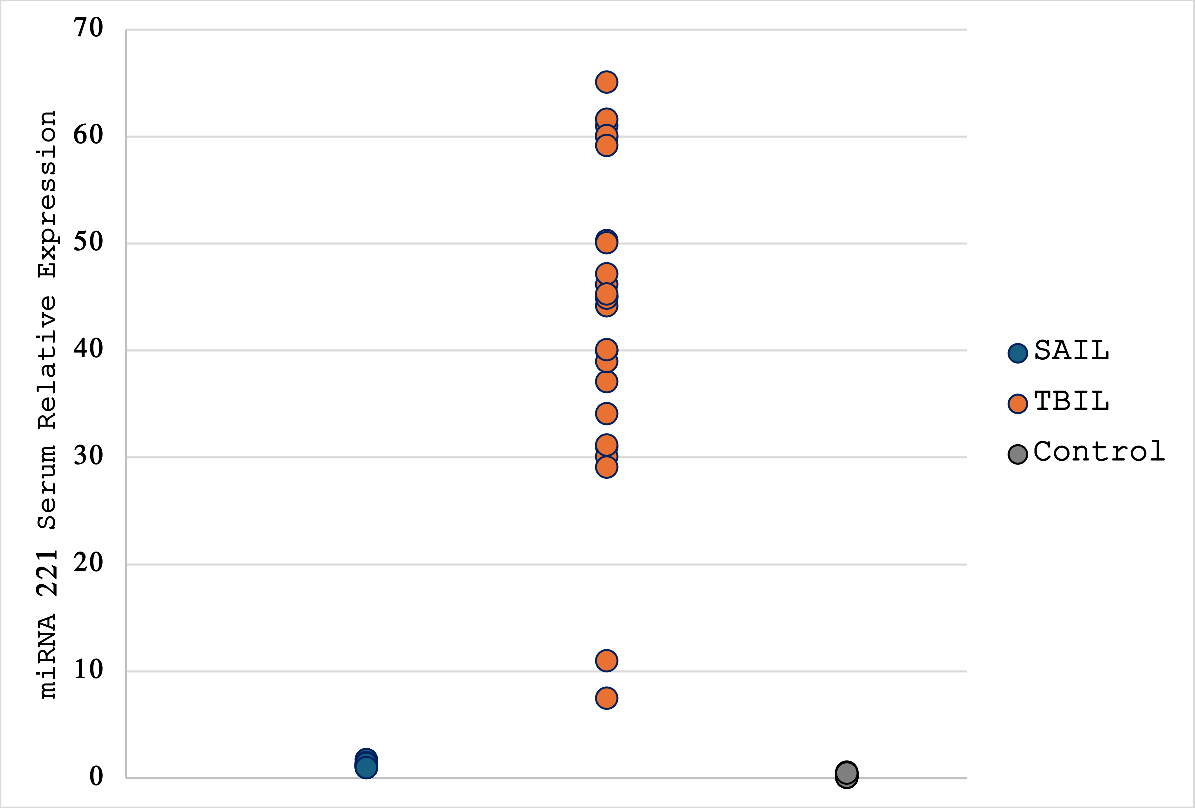

Supplementary Figure S2: Distributions of miRNA 221 Serum Relative Expression in patients with sarcoidosis-associated intrathoracic lymphadenopathy (SAIL), tuberculosis intrathoracic lymphadenopathy (TBIL), and healthy controls. Each dot represents one individual. Groups are color-coded for clarity (TBIL = blue, SAIL = orange, Control = gray).
